# Situating Biomedical and Professional Monopoly at the Intersections of Structural, Ideational and Agentic Power: Comment on "Power Dynamics Among Health Professionals in Nigeria: A Case Study of the Global Fund Policy Process"

**DOI:** 10.34172/ijhpm.2023.8019

**Published:** 2023-07-03

**Authors:** Anuj Kapilashrami

**Affiliations:** School of Health & Social Care, University of Essex, Colchester, UK

**Keywords:** Power, Global Health Initiatives, Global Health Partnerships, Health Systems, Health Policy

## Abstract

Lassa and colleagues’ study is a strong commentary on the biomedical hegemony and professional monopoly of medical doctors in the policy landscape of the Global Fund in Nigeria. Situating this critical dimension of professional power within wider scholarship of power and governance of global health initiatives (such as the Global Fund), in this comment, I put forth two core arguments. I call for a relational perspective of power in a dynamic policy space that the Fund characterises. I argue that a systems-view analysis of power requires a thorough examination of subsystems, how they interact, and the diverse forms of power—individual agentic, ideational, and structural—and the mechanisms through which power is wielded. The lens of governmentality allows linking individual (expertise and practices) with institutional regimes and social practices these enable; and in examining the interface of local/ sub-national, national, and global within which policy formulation and implementation occurs.

 The starting premise of Lassa et al^1^ is that policy failures result, in part, from the absence of ‘systems thinking’ and a multi-disciplinary approach that are necessary for problem solving. A core deterrent to the use of systems thinking the authors argue is the concentration of power in “the hands of a few actors, such as medical doctors,” and the hegemony of the biomedical paradigm that leads to policy failure. So, in order to uncover the “more disruptive effects” of power visible in policy failure, authors establish an urgency around generating a better understanding of various forms of power (and in effect its distribution and exercise). Such analysis of power is a well-acknowledged gap in studies examining global health institutions, especially those with significant influence in global agenda setting, albeit research on Global Fund to fight AIDS, tuberculosis and malaria (or Global Fund), GAVI and Gates Foundation from nearly a decade ago, including from the author, had at its heart the analysis of power and its distribution in the country-level and global governance of these initiatives.^2-6^

 Examining accounts of 34 in-country staff of organisations involved with Global Fund activities, Lassa et al reveal multiple sources of biomedical dominance in the policy process that establish the monopoly of medical profession in policy formulation and implementation within the Global Fund’s in-country governance. These include the dominance of medical or public health doctors in positions that drive the proposal writing and implementation process and their prominence in the Nigerian health system as well as in the international organisations (FHI-360) that constitute the civil society space in HIV/AIDS. Authors argue that medical doctors exercise this power and influence over Global Fund’s processes by virtue of their biomedical/public health training that places them in an authoritative position in relation to other stakeholders when considering evidence or developing policy solutions. This is enabled by the biomedical bias inherent in both the national health systems as well as critical governance mechanisms of the Fund such as the technical review panels. For instance, authors highlight preference for biomedical interventions, such as clinical testing and antiretroviral treatments, and epidemiological evidence that dominated in meetings discussing content of proposals and distanced stakeholders who did not have biomedical training. Authors also highlight the prescriptive nature of the Global Fund processes that made alignment to country roadmaps challenging, and the crucial role of consultants with medical expertise in proposal writing and achieving such alignment. Describing the grant application development processes within the Fund governance,^2,7^ I have previously highlighted how individuals regarded as having the ability to work in the context of extensive and complex application procedures became instrumental to successful bids and applications, and were hired as consultants to take on much of the grant preparation process. These studies examine practices of consultants and their embeddedness in elite networks but insufficiently interrogate disciplinary training as source of their professional power, which is a specific contribution of Lassa and colleagues’ study.

 In discussing the findings, the authors emphasise discursive power, and draw on the sociology of professions to examine these power dynamics among professionals. Drawing on sociological works of Freidson and Shiffman, they argue that professional monopoly of medical professionals is achieved through occupational hierarchy established through a colonial ‘professional bureaucrat model’ dominant in the public health sector in Nigeria. Such model places medical professionals at the helm of public (and private) sector institutions, and in the driving seat for agenda setting and policy development. The *productive* power exercised in deliberative processes is combined with the *structural* power through which Global Fund mechanisms invoke conformance and alignment with Global Fund priorities to maintain such professional monopoly and hierarchy.

 Although the paper does not investigate the various institutional and interpersonal consequences of such dominance, authors suggest that such assertive dominance in proposal writing potentially silences alternate views and expertise (for eg, social sciences and other disciplines or embodied knowledge of patients) and disregards or conceals the operational challenges. Notably, authors evidence low community uptake of medical supplies and resulting high wastage of resources (equal to 35 tons of expired HIV commodities at the central and state medical stores) documented in the audit reports. Elsewhere, I have illustrated how such dominance not only silences alternative views but co-opts and depoliticises them. Taking example of embodied movements such as networks of people living with HIV, I discussed how receiving funds from such global-local assemblages (characterising the Fund regime in India) undermines grassroots rights-focused advocacy, and reproduces mainstream discourses on technocratic ‘magic bullet’ solutions for complex socially determined health problems.

 Lassa et al study generates useful insights into professional (biomedical) hegemony within policy processes. However, there are few areas that warrant further attention and investigation.

 First relates to the relational aspect of power in a dynamic policy space such as the Fund governance and the national health *systems*. Previous assessments of these policy spaces reveal these as contested sites characterised by multiple and often conflicting interests, shifting allegiance, strategic brokering of alliances and resources, as well as of practices that effectively conceal power and sanitise narratives to align the reporting structures of the ‘protocol.’^2,4,8^ Such dynamic policy spaces establish the importance of seeing “sources of power as relational and context-dependent, rather than as fixed possessions or properties of actors”^9^ (p. 364) derived from technical and professional expertise alone. This context also calls for interrogating these sites for the resistance and friction created by the dominance of global and national elites and how civil societies, other professions and embodied health movements (for eg, people living with HIV networks) negotiate spaces, influence agendas, and are transformed in the process. Part of this contestation process is an ongoing negotiation of ideas around how problems and solutions are framed and presentation of these frames or narratives to both internal and external audiences to instigate desired action.^10^

 In examining health systems and policy spaces in Nigeria, Badejo et al^11^ report constant negotiations of professional roles and boundaries among health professionals despite the overall context of medical dominance. Not only did they find system disturbances or disequilibria resulting from such boundary struggles but also evidence of both conflictual and consensual shifts in professional power. They identify several facilitative conditions for the consensual shifts, including the possibility of simultaneous upward expansion of roles for all professions, introduction of new medical diagnostic technology that opened up occupational vacancies, among others. Resistance to medical dominance in Nigerian health systems is also noted by other scholars^12^ and evidenced in disputes between medical doctors and other health professionals resulting in strikes and formation of health sector unions that confront medical professions’ hegemony. How these resistances translate into the policy processes involving the Fund and the Nigerian health system remains unexplored in the paper. Furthermore, the notion and forms of expertise desired are constantly changing amid a changing environment defined by ongoing permeation of technologies and artificial intelligence and increasing managerialism in healthcare. Thus, management skills, knowledge of new diagnostics, data sciences and artificial intelligence have growing salience in health policy and may therefore be important sources of exercising professional power. These changing forms of expertise and how they shape professional power merit further examination.

 Second, a more nuanced understanding of discursive power and professional hegemony needs to be situated in the “complex and dialectical interaction between global actors and the nation state and between state and non-state actors” which is being “constantly negotiated, resisted and redefined at multiple levels”^13^ (p. 2). As previously argued, examining interactions between the global, the national and the ‘local’ allows an appraisal of “the multiple sites of power within which these relations are embedded, and the overt and covert ways in which global and national elites wield such power”^13^ (p. 2-3). The edited volume of research on global health governance in India enumerates several examples of overt and covert ways in which global health technical agencies and experts influence nutrition policies^14^ and maternal health programmes.^15^ In essence, the Nigerian health system and Global Fund governance that serve as the site for medical professionals’ dominance in policy-making examined in the study are themselves multi-tiered; wherein national and local actors from diverse sectors including public, businesses, and civil society are deploying Fund protocols to implement and monitor globally pre-determined priorities and approaches. They are therefore site of elite interactions that are constituted by diverse sources of power including but not restricted to professional power. Sriram et al^16^ identify several sources of power and their distribution in health policy and systems. Conceptualised primarily in relation to actors, these sources include power derived from: *technical* knowledge and their authoritative claim to that expertise, *political *authority and legitimacy, knowledge and access to *bureaucracies* and administrative machinery, access to *financial* capital, *personal* attributes including education and professional training, as well as *social capital* or access to networks and epistemic communities and the collective knowledge that comes with it. Examining the shifting inter-professional power relations shaped by the differential access to these sources helps understand actors’ agency as well as their *agentic* power, ie, their ability to act independently of the constraints of social structure.^17^

 Anthony Giddens proposed that power is clustered around the relations of action and structure.^18^ Here, the Foucauldian concept of governmentality can be useful for a fuller interrogation of power dynamics in health policy and systems. Governmentality goes beyond actors and formal institutions that govern to include “the organised practices, their rationalities, techniques, and protocols through which subjects are governed.”^19^ The concept helps bridge the structure-actor dichotomy in social theory that was challenged by Giddens and formed the basis of the structuration theory. As previously argued in the context of Global fund and the HIV/AIDS ecosystem in India, the concept allows investigation of the structures and institutions created within the Global Fund regime, their constitution and functioning, and the local-level practices these enable. In the context of the Fund, the Country Coordinating Mechanism, Technical Review Panel, and the Local Fund Agencies that act as ‘independent auditors’ of country performance tend to become key instruments of governmentality and exercise of *institutional *and *structural* power. Equally, a complex chain of Principal-Agent relationships of diverse actors becomes central to the Global Fund governmentality, as are “Fund brokers” in actively navigating these spaces and exercising *agentic* power. Aligned with Giddens’ structuration theory, this perspective views actors as ‘knowledgeable agents’ who are aware, to a great extent, about the conditions and consequences of their actions, and able to rationalise their actions/practices when needed. These agents act as ‘interpretive communities’ that help align the proposal content with external agendas and international principles and standards (as also observed by Lassa et al) alongside adapting its content to suit the interests of a diverse epistemic community of local and transnational actors. Through such acts of translation and adaptations they continually recruit support of diverse stakeholders, manage conflicting agendas, and demonstrate coherence and success by reproducing ideas (for eg, of participation, voice of people with HIV, as well as effectiveness of clinical and technical solutions) (*ideational* power*)*. Such act of translation demands a wider repertoire of expertise than that derived solely from biomedical expertise, and is critical to the reproduction of institutionalised practices.

 To summarise, as depicted in Figure, the Fund governmentality in country health systems is a complex and contentious policy space where power mediates the dynamic interactions between interacting global and local sub-systems. Here, practices are institutionally constituted and socially constructed by actors through an active process of translation and sensitisation of diverse expertise and interests. These actors or Fund brokers enable the practices of policy-making and its implementation to generate material outcomes and knowledge. The latter serves as a powerful tool in creating ‘order’ amid conflict and uncertainty, reproducing the dominant discourse on effectiveness of biomedical and other interventions and simultaneously legitimising the role of brokers and their practice. In this context, power operates at multiple levels and can be understood in terms of the networks, resources and expertise (eg, biomedical training) which actors leverage in their interactions to influence outcomes and agendas; the authority of institutional protocols and guidelines that shape perceptions and (dis)incentivise certain practices; and via social practices that embody power differentials and reproduce structures of domination (eg, exclusion from decision making and agenda setting mechanisms). Examining power thus demands going beyond professional and agentic power to unpack the role of hegemonic *structures* (systems and protocols) and *discourse* (ideas and meanings) in constituting and constructing practices that legitimise, give meaning and stabilise the fund and health system governance in Nigeria.

**Figure:**
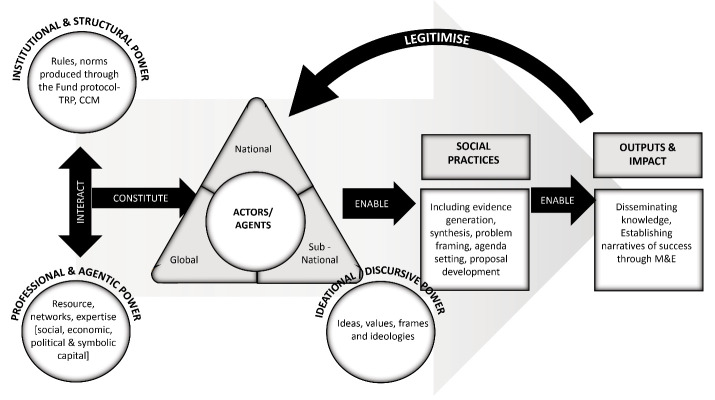
Sources and Interfaces of Power in Global Fund Governmentality. Abbreviations: TRP, Technical Review Panel; CCM, Country Coordinating Mechanism; M&E, monitoring and evaluation.

## Ethical issues

 Not applicable.

## Competing interests

 Author declares that she has no competing interests.
